# Closely related ST11 *Klebsiella pneumoniae* isolates with transient attenuation and re-emergence of carbapenem resistance during prolonged hospitalization: a case report

**DOI:** 10.3389/fimmu.2026.1857429

**Published:** 2026-10-02

**Authors:** Mingquan Zeng, Yu Jiang, Fei Yan, Yuting Sun, Heng Liao, Weiguo Zhou

**Affiliations:** 1 Department of Intensive Care Medicine, The Fourth People’s Hospital of Chengdu, Chengdu, China; 2 Department of Intensive Care Medicine, The Clinical Hospital of Chengdu Brain Science Institute, MOE Key Lab for Neuroinformation, University of Electronic Science and Technology of China, Chengdu, China; 3 Department of Clinical Laboratory, The Fourth People’s Hospital of Chengdu, Chengdu, China; 4 Department of Clinical Laboratory, The Clinical Hospital of Chengdu Brain Science Institute, MOE Key Lab for Neuroinformation, University of Electronic Science and Technology of China, Chengdu, China; 5 Department of Pharmacy, The Fourth People’s Hospital of Chengdu, Chengdu, China; 6 Department of Pharmacy, The Clinical Hospital of Chengdu Brain Science Institute, MOE Key Lab for Neuroinformation, University of Electronic Science and Technology of China, Chengdu, China

**Keywords:** carbapenem resistance, genomic relatedness, hypermucoviscosity, *Klebsiella pneumoniae*, KPC, phenotypic discordance, ST11

## Abstract

**Background:**

Apparent transitions in carbapenem susceptibility among sequential *Klebsiella pneumoniae* isolates are often interpreted as strain replacement. However, phenotypic variation among closely related isolates may also produce similar phenotypic shifts, particularly in heavily treated patients.

**Case presentation:**

We report a 91-year-old long-term hospitalized patient from whom three sequential ST11 *Klebsiella pneumoniae* isolates were recovered from blood and urine over a 3-month period. The initial bloodstream isolate (KPB1) exhibited a hypermucoviscous phenotype, KPC production, and carbapenem resistance. A subsequent urinary isolate (KPU2) lost the hypermucoviscous phenotype and showed attenuation of carbapenem resistance, becoming susceptible to imipenem, meropenem, and doripenem while remaining resistant to ertapenem. A later urinary isolate (KPU3) reverted to carbapenem resistance while remaining non-hypermucoviscous. Repeat antimicrobial testing confirmed these findings. Core-genome SNP analysis identified 16–20 pairwise differences among the three isolates, supporting close genomic relatedness despite their phenotypic discordance.

**Conclusion:**

This case illustrates marked differences in carbapenem susceptibility and hypermucoviscosity among closely related ST11 *K. pneumoniae* isolates recovered during a single patient’s clinical course. KPU2 retained ertapenem resistance despite susceptibility to other tested carbapenems. The genomic findings were more consistent with closely related isolates than with replacement by an unrelated strain, although direct ancestry and continuous persistence could not be established. Phenotypic changes should be interpreted alongside repeat microbiological testing, available genomic evidence, and the clinical context.

## 1 Introduction

Carbapenem-resistant *K. pneumoniae* (CRKP) is often discussed as a resistance category, but in clinical practice it is also a problem of interpretation. When recurrent isolates from the same patient show abrupt shifts in carbapenem susceptibility, carbapenemase phenotype, or colony morphology, such discordance may raise the possibility of strain replacement. This question is particularly relevant when an initial bloodstream isolate is followed by a urinary isolate with substantially reduced carbapenem resistance. Available evidence suggests that *K. pneumoniae* can exhibit considerable within-host genotypic and phenotypic diversity, meaning that such discordance does not necessarily indicate acquisition of an unrelated strain (1, 2). The clinical importance of ST11 *K. pneumoniae* lies in its high prevalence, multidrug resistance profile, and association with poor clinical outcomes in endemic settings (3).

This distinction is important because carbapenem resistance in *K. pneumoniae* is not always stable. Reversion from resistance to apparent susceptibility has been reported in KPC-producing strains (4). In addition, transferable plasmids can contribute to the dissemination and persistence of blaKPC-2 among Enterobacterales (5), while longitudinal studies have described plasmid dynamics during prolonged colonization (2, 11). In addition, permeability-related mechanisms, including porin alterations, may lead to intermediate or shifting carbapenem susceptibility patterns that complicate routine interpretation (6). Clinically, this means that an isolate appearing “susceptible” may represent either true strain replacement or transient remodeling within a persistent lineage.

A similar interpretive problem applies to mucoid phenotypes. Hypermucoviscosity is visually striking and often treated as if it were a stable organism-level trait, yet experimental and conceptual work has shown that hypermucoviscosity, capsule regulation, and hypervirulence are related but non-identical phenomena (7–9). Changes in mucoid appearance therefore should not be read too simply, especially when they occur alongside major shifts in resistance phenotype. When these features change together in sequential isolates, the combined effect can strongly suggest strain replacement even when the underlying lineage remains the same.

Against this background, we report three sequential ST11 *K. pneumoniae* isolates showing a resistant–attenuated–resistant carbapenem profile, accompanied by loss of hypermucoviscosity in the later isolates. Whole-genome sequencing was used to assess their genomic relatedness and provide descriptive context for the observed phenotypic differences.

## 2 Case presentation

### 2.1 Patient information and baseline clinical context

The patient was a 91-year-old man with prolonged hospitalization in a psychiatric specialty hospital. His medical background included cerebral atrophy, prior cerebral infarction, organic mental disorder, Alzheimer’s disease, parkinsonism, arrhythmia, severe pneumonia with type I respiratory failure and pleural effusion, prostatic hyperplasia with impaired urinary emptying, electrolyte disturbance, malnutrition-related anemia, and suspected gastrointestinal tumor. He had undergone tracheostomy more than 3 years earlier and required long-term enteral feeding through an indwelling gastric tube. Taken together, these conditions indicated a chronically vulnerable host with severe functional impairment, repeated healthcare exposure, and substantial cumulative antimicrobial pressure. The overall clinical course, major febrile episodes, urinary changes, antimicrobial exposure, and timing of recovery of the three principal isolates are summarized in **Figure 1**.

**Figure 1:**
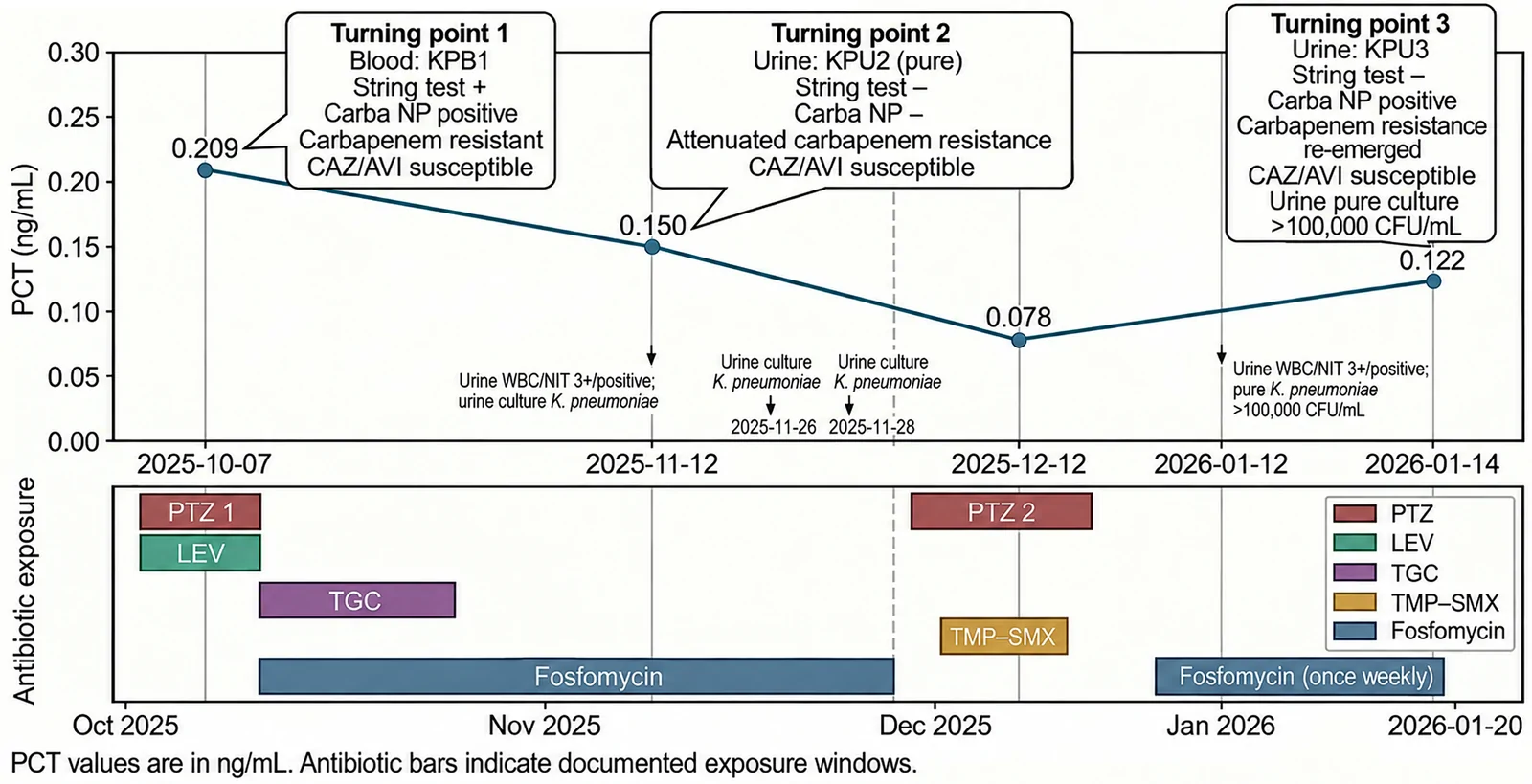
Clinical timeline of the three principal isolates. The image shows the dates of KPB1, KPU2, and KPU3 isolation, relevant fever and urinary findings, and documented antimicrobial exposure windows. Tigecycline was discontinued on November 23, 2025; piperacillin–tazobactam was administered from December 17 to December 23, 2025; fosfomycin trometamol was initially discontinued on December 12, 2025 and resumed once weekly from December 29, 2025 until January 14, 2026. KPU3 was isolated on January 12, 2026, while the intermittent fosfomycin regimen was still ongoing. Antibiotic bars indicate documented treatment periods and do not imply administration outside the recorded intervals.

### 2.2 Timeline and first infectious episode

The first decisive infectious episode occurred on October 7, 2025, when the patient developed high fever with a peak temperature of 39.5 °C. Blood culture from the aerobic bottle yielded *K. pneumoniae.* At that time, urinary abnormality was present but had not yet become the dominant clinical focus: the urine was described as turbid, urine WBC/NIT on October 10 was 1+/negative, and urine culture on October 13 showed no pathogenic growth. In parallel, inflammatory markers were elevated, including leukocytosis, neutrophilia, C-reactive protein, serum amyloid A, procalcitonin, and interleukin-6, supporting an active systemic infectious process.

The bloodstream isolate recovered on October 7 was designated KPB1. Species identification was performed by matrix-assisted laser desorption/ionization time-of-flight mass spectrometry using the Autobio MS1000 platform. Antimicrobial susceptibility testing was performed with the VITEK 2 system (bioMérieux), and carbapenemase phenotype was assessed using the Carba NP assay. KPB1 showed a hypermucoviscous colony phenotype, was string-test positive, Carba NP KPC-positive, and displayed resistance to the tested carbapenems. This first isolate therefore established the initial microbiological frame of the case as a bona fide bloodstream CRE-*K. pneumoniae* episode. Follow-up blood cultures on October 13 and October 16 were negative, and the fever gradually subsided during treatment.

### 2.3 Diagnostic assessment of the second episode

The second pivotal episode developed over the following month. From late October into early November, the urine changed from relatively clear to darker and then progressively turbid. On November 4, urine WBC/NIT became 3+/positive, indicating progression of urinary inflammation. On November 12, the patient again developed fever, ranging from 38.3 °C to 38.7 °C. Blood culture obtained that day was negative, whereas urine culture yielded *K. pneumoniae*, designated KPU2.

KPU2 was identified using the same MALDI-TOF workflow, and antimicrobial susceptibility testing was again performed using the VITEK 2 system, with repeat confirmation of decision-relevant results. In contrast to KPB1, KPU2 no longer displayed the same carbapenemase-associated microbiological pattern. The isolate lost the hypermucoviscous phenotype, became string-test negative, and was negative by Carba NP. Its carbapenem susceptibility profile was also attenuated compared with that of the initial bloodstream isolate: ertapenem remained resistant, whereas meropenem and doripenem became susceptible, and imipenem was reported as susceptible in the contemporaneous antimicrobial susceptibility record. At this point, the microbiological picture had shifted from the original bloodstream CRE pattern to a later urinary isolate with substantially reduced carbapenem resistance. This combination of changes made the second episode diagnostically important. The shift involved not only antimicrobial phenotype but also specimen source, moving from blood to urine while simultaneously losing the original CRE/KPC-associated profile. Read in real time, this pattern could readily support the impression that the initial high-risk strain had been replaced by a different urinary isolate with lower resistance. This pattern suggested a non-carbapenemase-mediated attenuation of carbapenem resistance rather than complete phenotypic reversion.

### 2.4 Therapeutic intervention and short-term response

After the first episode, the patient received empirical anti-infective treatment together with supportive care for documented bloodstream infection. The recorded regimen during this phase included piperacillin-tazobactam and levofloxacin initially, followed later by tigecycline and fosfomycin tromethamine, with additional symptomatic management such as physical cooling. Rather than representing a single fixed regimen, treatment was adjusted during the course according to the evolving clinical condition, microbiological findings, and bedside assessment. In the short term, the patient became afebrile and follow-up blood cultures turned negative, indicating temporary control of the initial bloodstream episode. Management of the second episode was interpreted in a different clinical setting. By mid-November, the dominant findings had shifted from bloodstream positivity to urinary abnormalities, and KPU2 no longer showed the original KPC-associated pattern. Antimicrobial treatment during this phase included tigecycline and fosfomycin-based therapy. The patient again showed short-term clinical improvement, but this did not establish a stable new microbiological state, as the later recurrence of urinary CRE indicated that the attenuation seen during the KPU2 phase was not durably maintained.

### 2.5 Follow-up, recurrent urinary CRE, and comparative microbiology

The third key episode forced the earlier interpretation to be reconsidered. In December 2025, the patient again developed nocturnal fever, reaching 39.5 °C on December 12. By January 12, 2026, the urine was turbid, urine WBC/NIT was 3+/positive, and urine culture yielded a pure growth of *K. pneumoniae* at >100,000 CFU/mL; this isolate was designated KPU3. At the time of KPU3 isolation, tigecycline and piperacillin–tazobactam had already been discontinued, whereas fosfomycin trometamol was being administered intermittently once weekly. Fosfomycin therapy was subsequently discontinued on January 14, 2026. The urinary isolates were interpreted as clinically significant infections rather than colonization, based on concurrent fever, progressive urinary inflammatory markers (urine WBC/NIT positivity), and clinical response following antimicrobial therapy.

KPU3 was identified using the same Autobio MS1000 MALDI-TOF workflow and underwent antimicrobial susceptibility testing with the VITEK 2 system, together with repeat confirmation of decision-relevant results and Carba NP testing. Unlike KPU2, this later urinary isolate no longer retained the attenuated carbapenem pattern. Instead, it reverted toward the resistant profile seen in the first episode: the isolate remained non-hypermucoviscous and string-test negative, but Carba NP again indicated KPC, and carbapenem resistance reappeared. Key agents that had appeared susceptible in KPU2, including meropenem and doripenem, returned to resistance.

To compare the microbiological sequence directly, the three principal isolates—KPB1, KPU2, and KPU3—were reviewed side by side. KPB1 was hypermucoviscous, string-test positive, Carba NP KPC-positive, and carbapenem resistant. KPU2 lost hypermucoviscosity, became string-test negative, was Carba NP negative, and showed reduced carbapenem resistance, with imipenem, meropenem, and doripenem interpreted as susceptible while ertapenem remained resistant. KPU3 remained non-hypermucoviscous and string-test negative, but again became Carba NP KPC-positive and reverted to carbapenem resistance. Thus, the dominant microbiological pattern across the three principal isolates was not progressive change in one direction, but a resistant–attenuated–resistant sequence.

The same trend was reflected in other selected agents. Cefoperazone–sulbactam remained resistant in all three isolates and therefore did not show the same temporal pattern as the carbapenems, whereas ceftazidime-avibactam, tigecycline, polymyxin B, and trimethoprim-sulfamethoxazole remained active throughout. The comparative microbiological features of the three principal isolates are summarized in **Table 1**, and the phenotypic discordance is shown in **Figure 2**.

**Table 1:** Comparative microbiological characteristics of the three principal isolates.

Characteristic	KPB1	KPU2	KPU3
Specimen/date	Blood/2025-10-07	Urine/2025-11-12	Urine/2026-01-12
String test	Positive	Negative	Negative
Colony phenotype	Hypermucoviscous	Non-hypermucoviscous	Non-hypermucoviscous
Carba NP	KPC positive	Negative	KPC positive
MLST	ST11	ST11	ST11
IPM	R (MIC≥16)	S (MIC = 0.25)	R (MIC≥16)
MEM	R (MIC≥16)	S (MIC = 1)	R (MIC≥16)
DOR	R (MIC≥8)	S (MIC = 0.5)	R (MIC≥8)
ETP	R (MIC≥8)	R (MIC = 4)	R (MIC≥8)
CAZ/AVI	S	S	S
TGC	S	S	S
PB	S	S	S
TMP-SMX	S	S	S
SCF	R	R	R

The table summarizes specimen source, key phenotypic features, carbapenemase testing, MLST type, and minimum inhibitory concentration (MIC) values for key carbapenems (imipenem, meropenem, doripenem, and ertapenem) in KPB1, KPU2, and KPU3. Antimicrobial susceptibility testing was performed using the VITEK 2 system (bioMérieux) and interpreted according to CLSI guidelines. Key carbapenem MIC results were further verified by E-test to ensure methodological consistency and accuracy. The most notable pattern was a transient attenuation of carbapenem resistance in KPU2, followed by re-emergence of the original resistant profile in KPU3.

**Figure 2:**
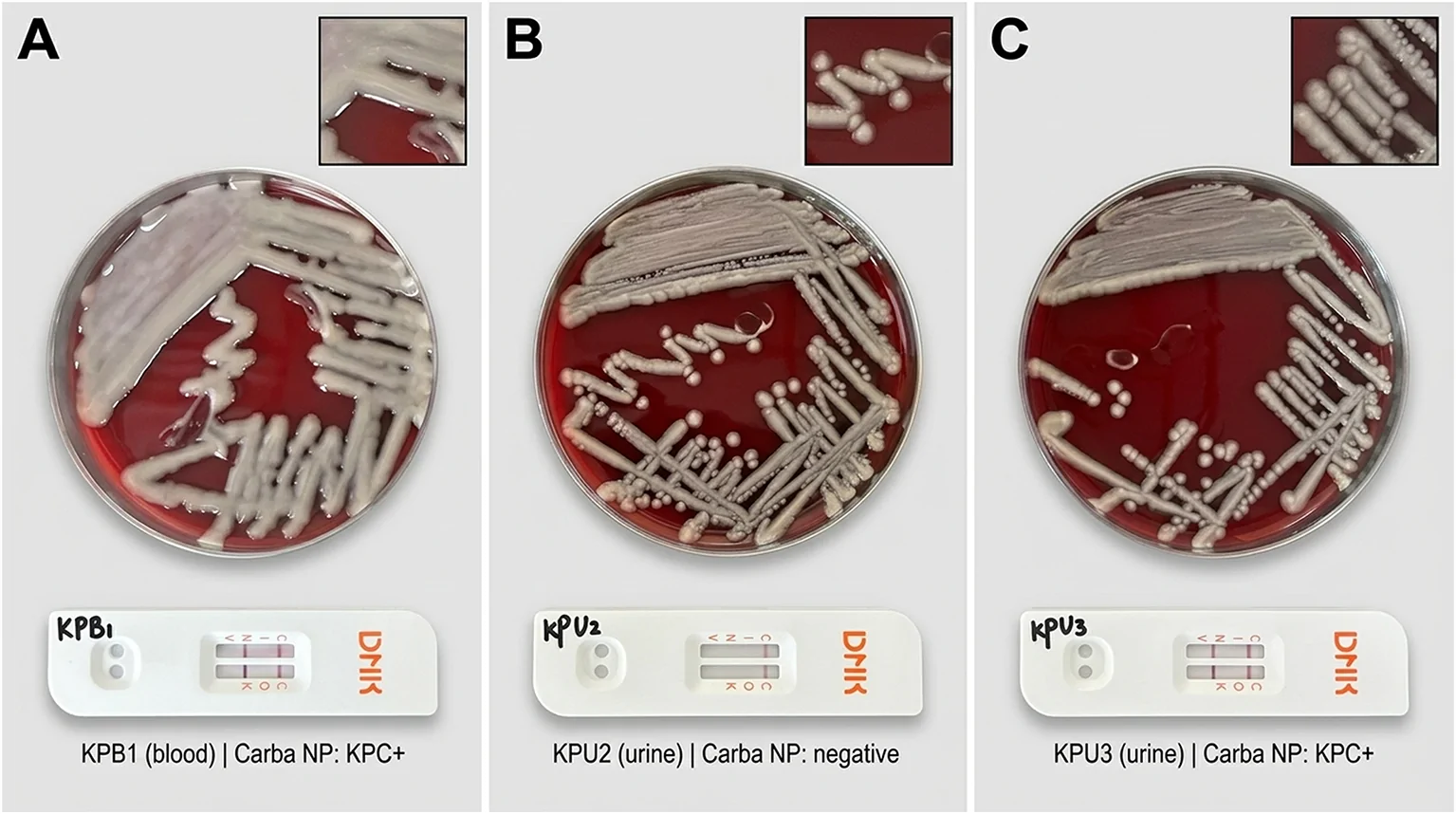
Phenotypic discordance across the three principal isolates. **(A)** Colony morphology under comparable culture conditions. KPB1 showed a hypermucoviscous appearance, whereas KPU2 and KPU3 did not. **(B)** String test results for the three isolates. KPB1 was string-test positive, while KPU2 and KPU3 were negative. **(C)** Carba NP results, showing KPC positivity in KPB1 and KPU3 but a negative result in KPU2. Together, these panels illustrate that the intermediate isolate differed markedly from both the initial and later isolates in visually and clinically relevant ways. All isolates were tested under identical laboratory conditions. String test interpretation followed standard criteria (>5 mm viscous string formation). Carba NP testing was performed using validated clinical microbiology protocols to determine carbapenemase activity.

### 2.6 Genomic adjudication

Whole-genome sequencing was performed to assess the genomic relatedness of KPB1, KPU2, and KPU3. Genomic DNA was extracted from pure cultures, and 300–500-bp PCR-free libraries were prepared for paired-end 150-bp sequencing on an Illumina NovaSeq 6000 platform. Raw reads were processed using fastp v0.20.1, and draft genomes were assembled *de novo* using SPAdes v3.13.0. Assembly quality and completeness were assessed using QUAST v5.0.2 and BUSCO v4.1.2, respectively. Genome annotation was performed using Prokka v1.14.6, and sequence types were assigned using mlst v2.19.0 against PubMLST. The complete KPU3 dataset contained 9,551,152 paired-end read pairs, corresponding to 19,102,304 reads and 2,865,345,600 bases. All reads were 150 bp in length. The combined raw-read Q20 and Q30 proportions were 96.50% and 94.08%, respectively. The R1 and R2 files contained identical numbers of reads, with matching read identifiers in the same order. These values represent unfiltered raw-read quality and are reported separately from the post-filtering quality metrics available for KPB1 and KPU2. Assembly statistics were recalculated from the existing draft FASTA files using a common minimum contig length of 500 bp. KPB1, KPU2, and KPU3 comprised 346, 158, and 142 contigs, with total assembly lengths of 5,720,769, 5,725,637, and 5,740,276 bp, respectively. The corresponding N50 values were 33,295, 97,344, and 94,397 bp, and the largest contigs were 96,928, 185,351, and 219,976 bp, respectively. These data are summarized in **Supplementary Table 2**. Reference-based variant analysis was performed for each isolate using Snippy v4.6.0 and the annotated HS11286 reference genome (GCF_000240185.1). Assembly-derived pseudo-reads were aligned with BWA-MEM v0.7.18, and alignment files were processed using SAMtools v1.20. Variants were called with FreeBayes v1.3.6 and filtered using BCFtools v1.21. The recorded filtering settings included a minimum mapping quality of 60, a minimum base quality of 13, and a minimum coverage of 10. Retained variants met the following criteria: GT = 1/1, QUAL ≥100, DP ≥10, and AO/DP ≥0.90. These thresholds were applied to the assembly-derived pseudo-read analysis and were not interpreted as measures of original sequencing depth or within-host allele frequency. Pairwise core-genome comparison showed 20 SNP differences between KPB1 and KPU2, 16 between KPB1 and KPU3, and 20 between KPU2 and KPU3, as shown in **Figure 3**. The corresponding distances from the HS11286 reference genome were 5,680, 5,684, and 5,668 SNPs, respectively. The within-case pairwise distances support close genomic relatedness among the three longitudinal isolates. However, these data do not establish a complete evolutionary pathway, uninterrupted persistence of every intermediate population, or the direction of any genomic change. Selected resistance- and virulence-associated gene-detection profiles were compared across the three draft genomes and are summarized in **Supplementary Table 1**. This targeted comparison was descriptive and was not used as evidence of gene expression or functional activity. Failure to detect a target in a draft assembly was therefore not interpreted as definitive evidence of gene deletion, plasmid loss, or structural rearrangement.

**Figure 3:**
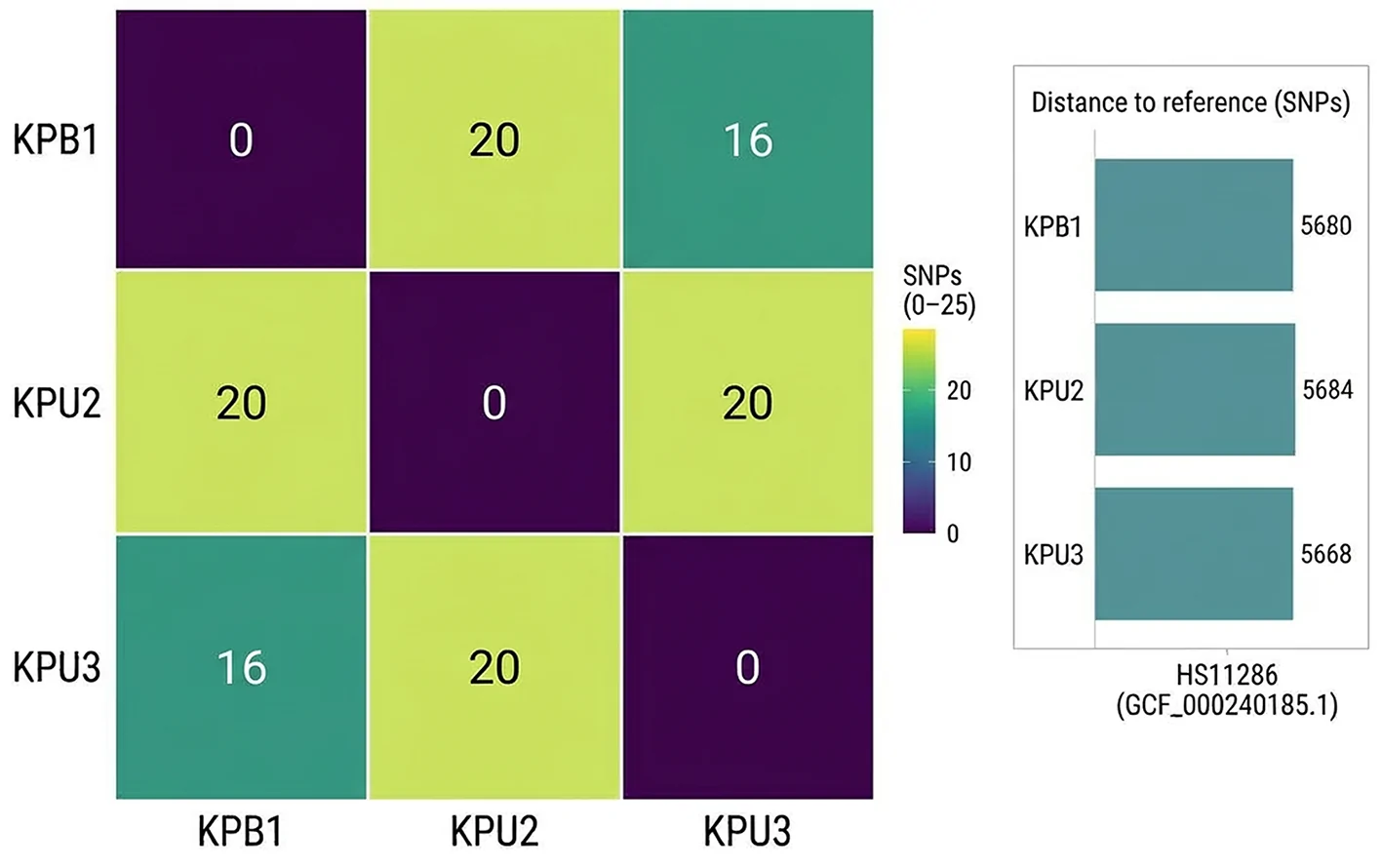
Pairwise core-genome SNP distances were 20 SNPs between KPB1 and KPU2, 16 SNPs between KPB1 and KPU3, and 20 SNPs between KPU2 and KPU3. The separate reference-comparison panel shows the distances of KPB1, KPU2, and KPU3 from HS11286 (GCF_000240185.1). The two panels represent different comparisons and should not be interpreted as a single phylogenetic scale. The results support close genomic relatedness among the three isolates but do not resolve chromosome or plasmid structure or establish the direction of evolutionary change.

## 3 Discussion

This case documents marked phenotypic discordance among three closely related ST11 *K. pneumoniae* isolates recovered from blood and urine during a single patient’s clinical course. The urinary isolate KPU2 differed from KPB1 in hypermucoviscosity, the reported carbapenemase result, and susceptibility to imipenem, meropenem, and doripenem. KPU3 subsequently showed a return to the more resistant carbapenem profile while remaining non-hypermucoviscous. These changes could raise the possibility of strain replacement. The 16–20 pairwise SNP differences support a highly related ST11 background, but close relatedness alone does not resolve the complete within-host history. The clinical contribution is the distinction between phenotypic discordance and evidence of replacement by an unrelated strain (1).

Cheng and colleagues demonstrated genotypic and phenotypic diversity among contemporaneous CRKP isolates from blood cultures of individual patients, indicating that a single colony or time point may not capture the full within-host population (1). Longitudinal studies have also described plasmid dynamics in KPC-positive *K. pneumoniae* during colonization (2, 11). These observations provide context for the present case, but do not establish that the same mechanism operated in this patient.

The intermediate isolate requires particular caution. Prior reports have described reversion toward carbapenem susceptibility in KPC-producing *K. pneumoniae* (4), and resistance can be influenced by resistance determinants, plasmid context, permeability, regulation, and population composition (2, 6, 11–14). In this case, *blaKPC* was not detected in the KPU2 draft genome. This finding does not establish transcriptional silencing, because transcriptomic measurements were not performed. The short-read assemblies did not permit definitive reconstruction of plasmid architecture or associated structural changes. Plasmid copy number was not assessed using a validated quantitative analysis, and transcriptional activity was not evaluated because gene-expression analyses were not performed. Genomic DNA sequencing does not directly measure transcription, regardless of read length. Although *ompK36* sequences were reported in the assemblies, their functional or expression-level contributions were not tested. Changes involving mobile elements, persistence of different subpopulations, and regulatory effects remain hypotheses, rather than demonstrated explanations for the observed sequence.

KPU2 did not become uniformly susceptible to carbapenems: ertapenem remained resistant while imipenem, meropenem, and doripenem were reported as susceptible. This distinction should be maintained when interpreting the clinical sequence. Porin-associated mechanisms have been investigated in studies of carbapenem resistance, including an *ompK36* variant associated with ertapenem resistance and altered *ompK36* translation (6, 12). However, these studies do not establish a porin mechanism in KPU2. The present findings describe attenuation of resistance, not conversion to a uniformly carbapenem-susceptible or non-CRE isolate.

The mucoid phenotype also warrants restraint. KPB1 was hypermucoviscous and string-test positive, whereas KPU2 and KPU3 were not. Hypermucoviscosity, capsule production, and hypervirulence are related but distinct properties (7–9). The observed change in specimen source coincided with the loss of hypermucoviscosity, but this temporal association does not demonstrate urinary-tract adaptation or selection. The targeted gene screen cannot establish capsule expression, hypervirulence, or the physical colocation of resistance and virulence determinants on a plasmid. Reports of resistance–virulence convergence provide broader biological context (10, 15), but no such structural mechanism is demonstrated here. The selected gene-detection differences and any subsequently validated SNP candidates require functional investigation before they can be linked to phenotype.

Within-host subpopulation structure is another possible explanation. Heteroresistance has been described in *K. pneumoniae* and can complicate interpretation of routine susceptibility testing (13, 14). We did not perform population analysis profiling or deep colony sampling, and therefore do not claim to have demonstrated heteroresistance. Three sequential isolates cannot distinguish the emergence of a new variant from changing recovery of pre-existing subpopulations. Likewise, pure growth in a urine culture does not establish that the entire within-host bacterial population was genetically homogeneous.

In clinical practice, a marked change in phenotype should prompt verification of decision-relevant susceptibility results and reassessment of the carbapenemase result, with close communication between the laboratory and treating team. Genomic comparison may help when the distinction between phenotypic change and strain replacement would materially affect interpretation. A susceptible result for selected carbapenems should not be equated with microbiological clearance or absence of carbapenem resistance when ertapenem remains resistant. Treatment decisions require the verified isolate-specific susceptibility profile and the contemporaneous clinical assessment, rather than an assumption of either persistence or replacement.

This case report has several limitations. Only three isolates from a single patient were available, and the sampling was insufficient to characterize the full within-host population. Although the isolates were closely related genomically, the available data cannot establish direct ancestry, uninterrupted persistence, or the precise sequence of evolutionary events. The genomic comparison was limited to selected resistance- and virulence-associated determinants. We did not perform a comprehensive accessory-genome analysis or a validated site-level assessment of the variants corresponding to the reported pairwise SNP differences. The genetic basis of the changes in carbapenemase detection, carbapenem susceptibility, and colony phenotype therefore remains uncertain. The assemblies were generated from short-read data. Differences in assembly contiguity, particularly the more fragmented KPB1 assembly, mean that failure to detect a gene may reflect incomplete sequence recovery or annotation rather than true gene loss. The current data cannot resolve complete chromosome or plasmid structures or demonstrate plasmid acquisition, loss, or rearrangement. Long-read sequencing, validated plasmid copy-number analysis, transcriptomic analysis, population analysis profiling, and functional experiments were not performed. Accordingly, possible contributions from structural variation, regulatory changes, porin-associated mechanisms, or population heterogeneity remain unconfirmed. Rectal surveillance cultures were not performed during hospitalization, and no rectal isolates were available for phenotypic or genomic comparison. The gastrointestinal tract could therefore not be evaluated as a potential reservoir of the ST11 lineage. The close genomic relatedness of the bloodstream and urinary isolates does not, by itself, identify an anatomical reservoir or distinguish continuous persistence from reintroduction of a closely related strain.

## 4 Conclusions

Three closely related ST11 *K. pneumoniae* isolates recovered during a single patient’s clinical course showed substantial differences in carbapenem susceptibility and hypermucoviscosity. The intermediate urinary isolate retained ertapenem resistance despite susceptibility to imipenem, meropenem, and doripenem, and the later isolate again exhibited the more resistant profile. The genomic findings support close relatedness but do not establish a specific mechanism of persistence, resistance change, or plasmid evolution. The clinical implication is that marked phenotypic differences should prompt careful interpretation of repeat microbiological findings and available genomic evidence rather than an automatic assumption of replacement by an unrelated strain.

## Data Availability

The whole-genome sequencing reads generated in this study have been deposited in the NCBI Sequence Read Archive under BioProject accession PRJNA1505938. Supporting clinical and microbiological data are presented in the article and its **Supplementary Material**. Additional non-identifying analysis files are available from the corresponding author upon reasonable request and in accordance with institutional requirements.
