# Supplementary material for: Closely related ST11 Klebsiella pneumoniae isolates with transient attenuation and re-emergence of carbapenem resistance during prolonged hospitalization: a case report
Source: Front Immunol. 2026 Oct 2;17:1857429. doi: 10.3389/fimmu.2026.1857429 (PMC13672232; doi:10.3389/fimmu.2026.1857429)
Supplement: Supplementary file 1 [file Table1.docx]

# Supplementary Table 1

Targeted comparison of selected resistance- and virulence-associated gene-detection profiles among KPB1, KPU2, and KPU3.

| **Gene or gene group** | **Functional annotation** | **KPB1** | **KPU2** | **KPU3** |
| --- | --- | --- | --- | --- |
| blaKPC | carbapenemase | Detected | Not detected | Detected |
| blaTEM-1B | β-lactamase | Detected | Detected | Detected |
| rmtB | 16S rRNA methyltransferase | Detected | Detected | Detected |
| tet(G) | tetracycline resistance | Detected | Not detected | Not detected |
| cmlA9 | phenicol resistance | Detected | Not detected | Not detected |
| qacEΔ1 | quaternary ammonium compound resistance-associated | Detected | Not detected | Not detected |
| mrk-associated genes* | type 3 fimbrial-associated | Detected | Detected | Detected |

* “mrk-associated genes” refers to the mrk genes included in the original targeted comparison; the table does not imply complete operon reconstruction or expression. “Not detected” means that the target was not identified by the applied screening workflow in the draft assembly and does not establish true gene absence, deletion, or plasmid loss. The table is descriptive and does not constitute functional validation.

# Supplementary Table 2

Raw-read quality metrics for KPU3 and assembly statistics for KPB1, KPU2, and KPU3.

| **Assembly metric** | **KPB1** | **KPU2** | **KPU3** |
| --- | --- | --- | --- |
| Contigs ≥500 bp | 346 | 158 | 142 |
| Total assembly length (bp) | 5,720,769 | 5,725,637 | 5,740,276 |
| N50 (bp) | 33,295 | 97,344 | 94,397 |
| Largest contig (bp) | 96,928 | 185,351 | 219,976 |
| L50 | 57 | 23 | 23 |
| GC content (%) | 57.06 | 57.05 | 57.05 |

Assembly statistics were recalculated from the existing contig FASTA files using a common minimum contig length of 500 bp. N50 is the contig length at which cumulative contig length, ordered from longest to shortest, first reaches at least 50% of total assembly length. No N bases were present in the analyzed FASTA records.

KPU3 raw-read metrics

| **Metric** | **KPU3 combined R1+R2** |
| --- | --- |
| Read pairs | 9,551,152 |
| Total reads | 19,102,304 |
| Total bases (bp) | 2,865,345,600 |
| Read length (bp) | 150 per mate |
| Q20 bases (%) | 96.50 |
| Q30 bases (%) | 94.08 |
| GC content (%) | 54.55 |
| N bases | 139,055 |

KPU3 metrics were calculated from the complete raw FASTQ files using Phred+33 quality scores. R1 and R2 contained identical read counts and matching read identifiers in the same order. Q20 and Q30 are the proportions of all raw bases with Phred scores ≥20 and ≥30, respectively. These are raw-read values before read filtering and are therefore reported separately from the post-filtering CleanQ30 values previously available for KPB1 and KPU2. Raw-read metrics should not be interpreted as mean alignment depth or as evidence of sample purity.
